# Effect of Procyanidin on Canine Sperm Quality during Chilled Storage

**DOI:** 10.3390/vetsci9110588

**Published:** 2022-10-26

**Authors:** Xiaogang Huang, Zhihong Zhao, Ronggen Wang, Ying Ma, Yonghui Bu, Minhua Hu, Shouquan Zhang

**Affiliations:** 1 National Engineering Research Center for Breeding Swine Industry, College of Animal Science, South China Agricultural University, Guangzhou 510642, China; 2 Guangdong Province Key Laboratory of Molecular Breeding, College of Animal Science, South China Agricultural University, Guangzhou 510642, China; 3 National Canine Laboratory Animal Resources Center, Guangzhou General Pharmaceutical Research Institute Co., Ltd., Guangzhou 510240, China

**Keywords:** procyanidin (PC), chilled storage, canine sperm, sperm quality

## Abstract

**Simple Summary:**

In recent years, artificial insemination (AI) using chilled sperm has been one of the most utilized artificial assisted reproductive technologies for dogs. However, changes in temperature can cause oxidative stress, damage sperm structure, and even lead to sperm death. Procyanidin is a polyphenolic compound with antioxidant activity. This study was designed to investigate the effect of procyanidin on canine sperm quality when stored at 4 °C for 72 h. Procyanidin presented improvements in sperm motility, plasma membrane integrity, acrosome integrity, and mitochondrial membrane potential after being stored at 4 °C for 72 h, as well as total antioxidant capacity (T-AOC) and the expression levels of antioxidant-related genes. It could be concluded that the supplementation of procyanidin in the extender improved sperm quality when stored at 4 °C, and the antioxidant capacity of procyanidin could be an important factor in the improvement of sperm quality.

**Abstract:**

Procyanidin (PC) is a polyphenolic compound with antioxidant activity. The purpose of this study was to determine the influence of PC on canine sperm quality after 72 h of storage at 4 °C. The collected ejaculates were separated into four equal aliquots and treated with various concentrations of PC (0, 10, 30, and 50 μg/mL) in Tris-citric-fructose-egg yolk (TCFE) extender and stored at 4 °C for 72 h. The findings revealed that 30 μg/mL PC was the optimum concentration for significantly improving sperm motility (*p* < 0.05). Sperm samples treated with 30 μg/mL PC had substantially greater plasma membrane integrity, acrosome integrity, and mitochondrial membrane potential than the control group (*p* < 0.05). Furthermore, T-AOC and the expression levels of superoxide dismutase 1 (SOD1), catalase (CAT), and glutathione peroxidase 1 (GPx1) genes were significantly higher in sperm treated with 30 μg/mL PC than those in control (*p* < 0.05). In summary, this study discovered that adding PC to the TCFE extender enhanced sperm quality and that 30 μg/mL PC was the optimal concentration for canine sperm when stored at 4 °C.

## 1. Introduction

The Beagle dogs are internationally recognized experimental dogs and are widely used in biological and medical research, especially in non-clinical drug research. With the development of the domestic and foreign pharmaceutical industry, the demand for fine experimental Beagle dogs is also increasing. Therefore, it is urgent to carry out the conservation of high-quality Beagle dogs. Semen preservation is an important technique of in vitro conservation of male dogs, including chilled preservation (4 °C~5 °C) and cryopreservation (liquid nitrogen) [1]. In recent years, artificial insemination (AI) using chilled sperm has emerged as one of the most utilized artificial assisted reproduction methods for dogs [2]. In addition, studies have shown that chilled sperm have higher pregnancy rates and litter size than cryopreserved sperm [3].

However, sperm is very sensitive to changes in temperature. Due to changes in the internal and exterior environment of cells, sperm will create extra reactive oxygen species (ROS) throughout the cooling process. The excess ROS carrying highly oxidative radicals can trigger lipid peroxidation in the sperm plasma membrane, leading to oxidative stress. As a result, the sperm plasma membrane and acrosome structure will be damaged, sperm motility will be reduced, and sperm death may occur [4,5]. Therefore, in order to reduce the oxidative damage of sperm during chilled preservation, different antioxidants are usually added to extenders to improve the antioxidant capacity of sperm, including vitamin E, vitamin B16 [6], glutathione [7,8], and lycopene [9].

Procyanidin (PC) is a polyphenolic compound found in plants that exhibits considerably greater antioxidant activity than vitamin C and vitamin E [10], allowing it to effectively eliminate superoxide anion radicals and hydroxyl radicals, while also improving the antioxidant enzyme activity [11]. In addition, it is also involved in the metabolism of phospholipids and arachidonic acid and the phosphorylation of proteins [12], reducing the degree of cellular lipid peroxidation [13] and reducing cellular oxidative damage [10,14,15,16]. Studies have indicated that procyanidin enhanced the incubated or post-thaw sperm quality of human [17], boar [18], goat [19], hu sheep [20], and bull [21]. However, there is no report to date on the effects of procyanidin on dog sperm chilled preservation.

The objective of this study was to look into the influence of procyanidin on the quality of sperm preserved at 4 °C. Sperm motility, kinetic parameters, plasma membrane integrity, acrosome integrity, mitochondrial membrane potential, total antioxidant capacity (T-AOC), and gene expression were studied.

## 2. Materials and Methods

### 2.1. Chemicals and Sources

The procyanidin used in this study was purchased from Macklin (Shanghai Macklin Biochemical Co., Ltd., Shanghai, China), and other chemicals used in this work were all purchased from Sigma-Aldrich unless indicated otherwise. The Tris-citric-fructose (TCF) extender used to wash and dilute canine sperm was composed of Tris 2.4 g, citric acid 1.4 g, fructose 0.8 g, kanamycin sulfate (Beijing Dingguo Changsheng Biotechnology Co., Ltd., Beijing, China) 0.015 g, and distilled water 100 mL [22]. The buffer (TCFE) used for canine sperm preservation was composed of 60% TCF (*v*/*v*) and 40% egg yolk (*v*/*v*).

### 2.2. Animals and Semen Collection

In this research, six Beagle dogs aged from two to four years were utilized. The dogs were stud dogs of Guangzhou General Pharmaceutical Research Institute Co., Ltd. (National Canine Laboratory Animal Resources Center, Guangzhou, China). The animals were in good health and normal reproductive condition, and they were raised in the kennel and fed dry food once a day, with free access to water. Manual masturbation was used to collect the semen from each dog once a week. The sperm-rich second portion of ejaculate was collected in a sterile tube with a water-coat pre-warmed to 38 °C.

### 2.3. Sperm Processing

All ejaculates were analyzed using phase-contrast microscopy (Eclipse Ti2, Nikon, ×200), and ejaculates with ≥70% motility, ≥80% viability, and ≥100 × 10^6^ sperm/mL concentration were pooled together (at least three ejaculates). To eliminate cellular debris, the pooled ejaculates were centrifuged at 100× *g* for 1 min at 38 °C. The supernatant was gathered in a newly sterile 15 mL tube with a water-coat pre-warmed to 38 °C followed by an equal volume of TCF. The seminal plasma was discarded via centrifuging at 700× *g* for 5 min at 38 °C, and the sperm pellet was resuspended using TCF to reach a sperm concentration of 200 × 10^6^ sperm/mL.

The resuspended sperm was separated into four equal aliquots and diluted with TCFE to reach a sperm concentration of 100 × 10^6^ sperm/mL supplemented with a final concentration of 0 (control), 10, 30, and 50 ug/mL PC. The extended semen samples were then cooled to 4 °C and refrigerated for 72 h. To avoid precipitation, the samples were gently shaken twice a day during storage.

Before the assessment of sperm quality, each sample was taken from the refrigerator and incubated in a water bath at 38 °C for 5 min.

### 2.4. Sperm Motility and Kinetic Parameters Analysis

Sperm motility and kinetic parameters were evaluated using a computer-assisted sperm analysis (CASA) system (WEILI Color Sperm Quality Analysis System version 5.0, WeiLi, Beijing, China). Briefly, 5 μL of the incubated semen was put in a disposable analysis chamber slide (ML-CASA10-4, Mailang, Nanning, China) and maintained at 38 °C. At least 200 sperm in 5 random fields were monitored to measure parameters including sperm total motility (TM), progressive motility (PM), curvilinear velocity (VCL), straight-line velocity (VSL), average path velocity (VAP), linearity (LIN), wobble coefficient (WOB), and straightness (STR). Each group included at least three replicates.

### 2.5. Sperm Plasma Membrane Integrity

**Figure 1:**
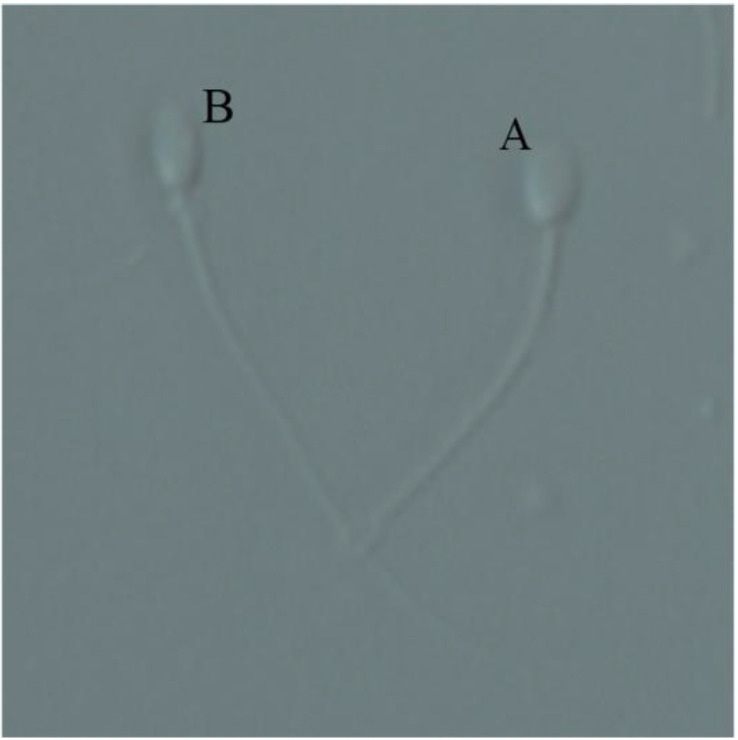
Assessment of sperm plasma membrane integrity using hypo-osmotic swelling test (HOST). Sperm with the coiled tail (A) considered membrane intact; those with the straight tail (B) considered membrane damaged (×200 Magnification).

Sperm plasma membrane integrity was assessed using a hypo-osmotic swelling test (HOST). Briefly, 10 μL of the incubated semen was combined with 100 μL HOST solution (0.735 g sodium citrate and 1.351 g fructose added to 100 mL distilled water) and incubated for 30 min at 38 °C. Following incubation, 10 μL of the mixture was put on a glass slide mounted with a coverslip, and at least 200 sperm were examined using a phase-contrast microscope (Eclipse Ti2, Nikon, ×200, Japan). The sperm with coiled tails were considered membrane intact (Figure 1). The percentage of sperm with intact membranes was calculated. Each group included at least three replicates.

### 2.6. Sperm Acrosome Integrity

**Figure 2:**
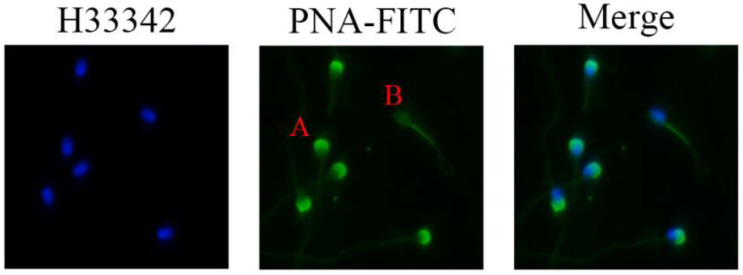
Assessment of sperm acrosome membrane integrity using fluorescein isothiocyanate-conjugated peanut agglutinin (FITC-PNA). Sperm with strong green fluorescence (A) considered acrosome intact; those with no fluorescence (B) considered acrosome damaged (×200 Magnification).

Sperm acrosome integrity was assessed using fluorescein isothiocyanate-conjugated peanut agglutinin (FITC-PNA). Briefly, 30 μL of the incubated semen was smeared on a glass slide and air-dried. The smears were fixed in anhydrous methanol at room temperature for 10 min before being incubated with FITC-PNA at 37 °C for 30 min under moist and dark conditions. The nuclei were stained with H33342. After incubation, the smears were washed three times with phosphate-buffered saline (PBS) for 5 min before being mounted with a coverslip. At least 200 sperm were examined using a fluorescence microscope (Eclipse Ti2, Nikon, ×200). The sperm with bright green fluorescence were considered to possess an intact acrosome (Figure 2). The percentage of sperm with intact acrosome was calculated. Each group included at least three replicates.

### 2.7. Sperm Mitochondrial Membrane Potential

**Figure 3:**
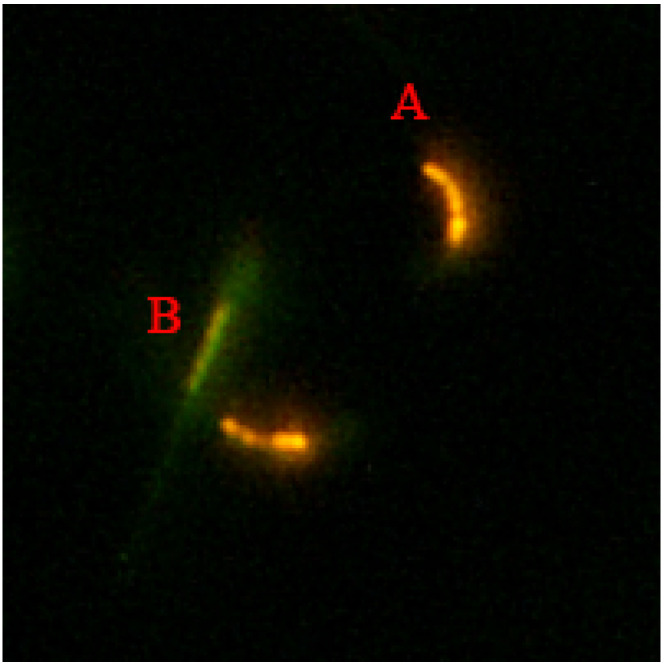
Assessment of sperm mitochondria membrane potential using JC-1. Sperm with red fluorescence (A) considered as with a high mitochondrial membrane potential; those with green fluorescence (B) considered as with low mitochondrial membrane potential (×200 Magnification).

Sperm mitochondrial membrane potential was evaluated using a Mitochondrial membrane potential assay kit with JC-1 (Beyotime Institute of Biotechnology, Shanghai, China). Briefly, the incubated semen was stained with 1 × JC-1 at 38 °C for 20 min under dark conditions. Following incubation, the sample was washed three times with 1 × JC-1 dyeing buffer at 700× *g* for 5 min at 4 °C. After washing, 10 μL of the mixture was put on a glass slide mounted with a coverslip, and at least 200 sperm were examined using a fluorescence microscope (Eclipse Ti2, Nikon, ×200). The JC-1 monomer fluoresced green at low mitochondrial membrane potential, while the aggregates fluoresced red fluorescence at high mitochondrial membrane potential (Figure 3). The mitochondrial membrane potential of sperm in each group was determined as the red-to-green fluorescence ratio. Each group included at least three replicates.

### 2.8. Measurement of Sperm T-AOC

Sperm T-AOC was measured using the Total Antioxidant Capacity Assay Kit with a Rapid ABTS method (Beyotime Institute of Biotechnology, Shanghai, China). Briefly, the incubated semen was rinsed three times with PBS and resuspended before being lysed ultrasonically (20 kHz, 300 W, operating at 100%, on 3 s, off 5 s, 7 cycles) on ice and centrifuged at 12,000× *g* for 10 min at 4 °C. The supernatants were collected, and sperm T-AOC was measured according to the manufacturer’s procedure by a spectrophotometer (Spectrophotometer 1510, Thermo Fisher Scientific, Waltham, MA, USA) at 414 nm. Quantification of the extracted sperm proteins was measured by spectrophotometer at 562 nm using BCA Protein Assay Kit (KeyGEN BioTECH, Nanjing, China). T-AOC of each sperm sample was converted into mmol per g of total protein in spermatozoa and expressed as mmol/g. Each group included at least three replicates.

### 2.9. Gene Expression Analysis

Quantitative polymerase chain reaction (qPCR) was used to analyze the mRNA expression of genes related to antioxidant (superoxide dismutase 1, SOD1; catalase, CAT; and glutathione peroxidase 1, GPx1). Real-time qPCR (RT-qPCR) was performed to assess transcript abundance using primer sequences listed in Table 1. Briefly, total RNA was extracted using FastPure Cell/Tissue Total RNA Isolation Kit (Vazyme Biotechnology Co., Ltd., Nanjing, China), followed by the synthesis of complementary DNA (cDNA) using HiScript II 1st Strand cDNA Synthesis Kit (Vazyme Biotechnology Co., Ltd., Nanjing, China) following the manufacturer’s protocol. ChamQ Universal SYBR qPCR Master Mix (Vazyme Biotechnology Co., Ltd., Nanjing, China) was used to assess expression levels of RT-qPCR transcripts and the expression of each target gene was quantified relative to that of the internal gene (actin beta, ACTB) using the equation, R = 2^−[∆Ct sample − ∆Ct control]^. Each group included at least three replicates.

### 2.10. Statistical Analysis

Statistical Product Service Solutions version 26.0 (SPSS Inc., Chicago, IL, USA) and GraphPad Prism 8.0 (La Jolla, CA, USA) were used to analyze the data. One-way analysis of variance (ANOVA) and Tukey’s multiple comparison tests were used to analyze the data related to sperm motility, kinetic parameters, membrane integrity, and acrosome integrity. The independent sample t test was used to analyze the data relating to T-AOC and expression levels of the different genes. All values were expressed as mean ± standard error of the mean (SEM), and differences with *p*-value < 0.05 were considered to be statistically significant.

## 3. Results

### 3.1. Effects of Procyanidin on Sperm Motility and Kinetic Parameters

**Figure 4:**
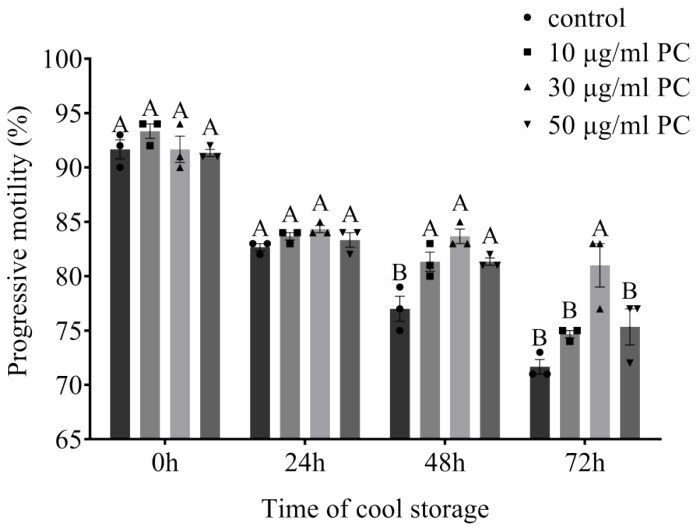
Effects of different concentrations of procyanidin on dog sperm progressive motility after being stored at a cool temperature for 72 h. Values are expressed as mean ± SEM (*n* = 3). Different uppercase superscripts (A, B) at the same time indicate significant differences (*p* < 0.05).

The effects of procyanidin on sperm motility and kinetic parameters evaluated at 0, 24, 48, and 72 h by CASA are shown in Figure 4 and Table 2. The values of TM and PM decreased during the storage time for all groups. At 0 h, there was no significant difference in TM and PM between treatment (10, 30, 50 μg/mL PC) and control groups, nor in ALH. However, the VCL, VSL, and VAP of samples treated with 10 μg/mL PC were lower than those of other groups (*p* < 0.05). At 24 h, TM was higher in treatment groups than in the control group (*p* < 0.05), but there was no significant difference in PM or ALH across all groups. VCL, VSL, and VAP were higher in the control group than in the treatment groups (*p* < 0.05). At 48 h, TM and PM were higher in the treatment groups than in the control group, as well as LIN, WOB, STR, and ALH (*p* < 0.05). Samples treated with 30 μg/mL PC showed the highest values of TM, PM, and ALH. At 72 h, samples treated with 30 μg/mL PC had higher TM, PM, and ALH levels than other groups (*p* < 0.05). Furthermore, the values of TM and PM in samples treated with 30 μg/mL PC showed no significant decrease during 24–72 h storage.

### 3.2. Effects of Procyanidin on Sperm Plasma Membrane Integrity

The effects of procyanidin on sperm plasma membrane integrity evaluated at 0, 24, 48, and 72 h by HOST are displayed in Table 3. The results indicated that the percentage of sperm with intact membranes decreased significantly with time for all groups (*p* < 0.05). At 0 h, the membrane integrity appeared to be significantly improved by being treated with 30 μg/mL PC (*p* < 0.05). At 24, 48, and 72 h, the membrane integrity of sperm treated with PC was significantly greater compared with the control group (*p* < 0.05) and was greatest at 30 μg/mL PC groups.

### 3.3. Effects of Procyanidin on Sperm Acrosome Integrity

The effects of procyanidin on sperm acrosome membrane integrity evaluated at 0, 24, 48, and 72 h by FITC-PNA are displayed in Table 3. There was no significant difference in sperm acrosome integrity between the treatment and control groups at 0 h. The sperm acrosome integrity was significantly decreased from 24 h to 72 h for all groups (*p* < 0.05), while PC protected sperm acrosome integrity, with PC at 30 μg/mL dramatically improving sperm acrosome integrity compared with the control group at 24 h to 72 h (*p* < 0.05).

### 3.4. Effects of Procyanidin on Sperm Mitochondrial Membrane Potential

**Figure 5:**
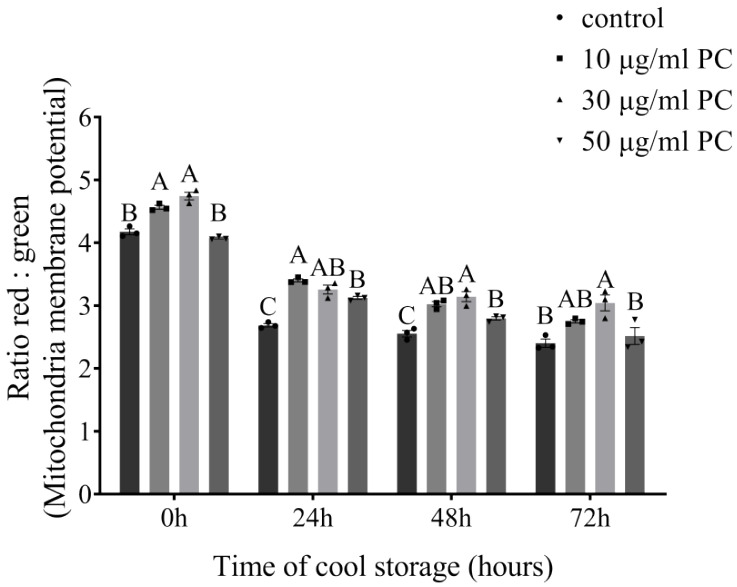
Effects of different concentrations of procyanidin on dog sperm mitochondrial membrane potential after being stored at a cool temperature for 72 h. Values are expressed as mean ± SEM (*n* = 3). Different uppercase superscripts (A, B, and C) at the same time indicate significant differences (*p* < 0.05).

The effects of procyanidin on sperm mitochondrial membrane potential evaluated at 0, 24, 48, and 72 h by JC-1 are displayed in Figure 5. The sperm mitochondrial membrane potential was significantly higher after being treated with procyanidin compared with the control group from 0 h to 48 h (*p* < 0.05), while only PC at 30 μg/mL significantly improved the mitochondrial membrane potential at 72 h (*p* < 0.05).

### 3.5. Effect of Procyandin on Sperm T-AOC

**Figure 6:**
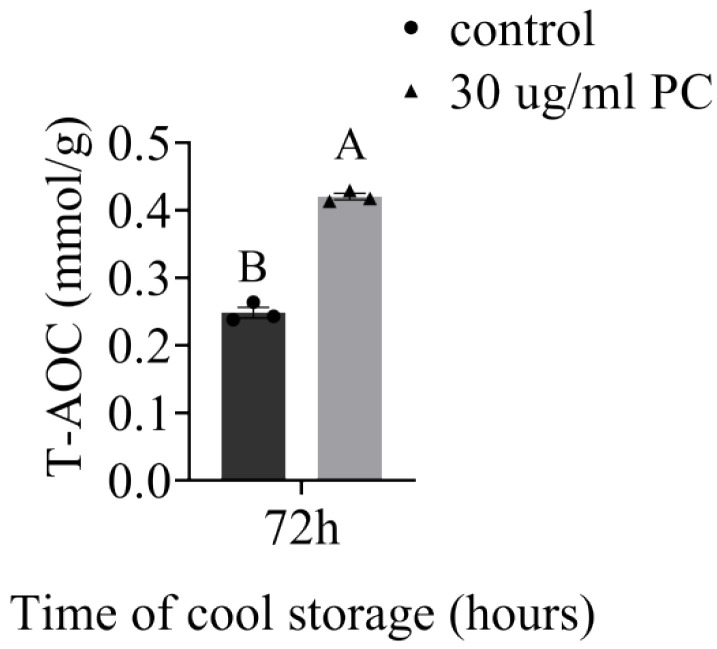
T-AOC of control samples and procyanidin-supplemented dog sperm samples after being stored at a cool temperature for 72 h. Values are expressed as mean ± SEM. Different uppercase superscripts (A and B) indicate significant differences (*p* < 0.05).

T-AOC of sperm samples without or with 30 μg/mL PC was measured at 72 h, and the results are displayed in Figure 6. The sperm T-AOC was significant higher after being treated with 30 μg/mL PC than in the control group (*p* < 0.05).

### 3.6. Effects of Procyandin on Gene Expression

**Figure 7:**
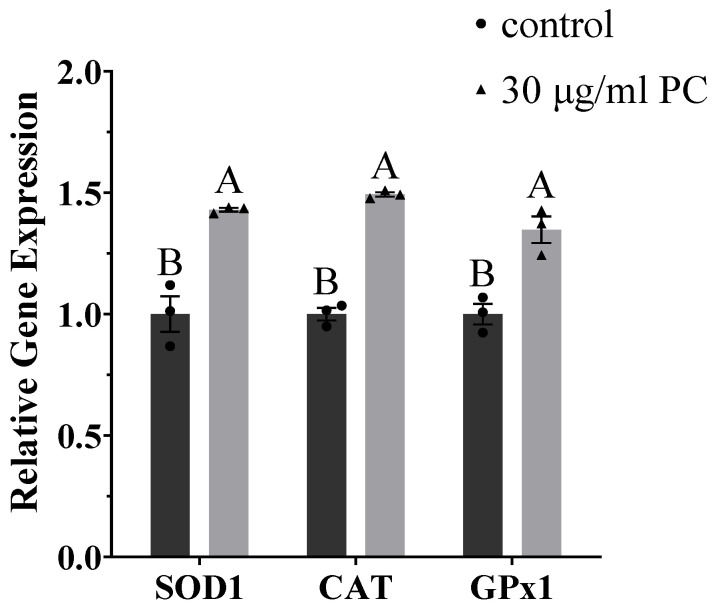
Expression levels of the superoxide dismutase 1 (SOD1), catalase (CAT), and glutathione peroxidase 1 (GPx1) genes in control samples and procyanidin-supplemented dog sperm samples using real-time quantitative polymerase chain reaction (RT-qPCR). Values are expressed as mean ± SEM. Different uppercase superscripts (A and B) indicate significant differences (*p* < 0.05).

The gene expression of sperm samples without or with 30 μg/mL PC was evaluated at 72 h, and the results are displayed in Figure 7. The findings indicated a significant increase in the expression levels of superoxide dismutase 1 (SOD1), catalase (CAT), and glutathione peroxidase 1 (GPx1) genes in the sperm treated with 30 μg/mL PC compared with the control group (*p* < 0.05).

## 4. Discussion

In a normal physiological state, the oxidation and antioxidant in sperm are in a state of dynamic balance. Sperm produces low levels of ROS during normal metabolism. Physiological levels of ROS help sperm mature in the epididymis, maintain sperm motility and increase membrane fluidity [23,24,25,26]. However, during the process of chilling, the activity of antioxidant enzymes in sperm will be reduced, and in the meantime, dead and damaged sperm will produce more ROS, resulting in an imbalance between sperm oxidation and antioxidant and excessive ROS accumulation in sperm. Excessive ROS will attack the polyunsaturated fatty acids on the sperm plasma membrane, causing lipid peroxidation of the plasma membrane, impairing its fluidity and ion permeability, disrupting its receptors and downstream signaling pathways, and ultimately disrupting membrane integrity and reducing sperm motility [27,28]. Similarly, excessive ROS will attack the acrosome membrane, resulting in the premature release of acrosome enzymes and failure of acrosome reaction, which ultimately reduces the fertility of sperm [29]. In addition, excess ROS will also attack mitochondria and reduce their membrane potential, resulting in a decrease in the efficiency of ATP production and ultimately affecting sperm motility [30,31].In recent years, there have been reports about adding different antioxidants to the extenders for chilled storage of dog sperm [6,7,8,9,32], but their addition has not been promoted, which may be due to their role in the preservation of different breeds of dog semen has not been verified.

Procyanidin is a powerful natural antioxidant derived from plants, which can effectively eliminate free radicals [11]. In this study, the effects of procyanidin on sperm quality were investigated after 0, 24, 48, and 72 h storage at 4 °C. The results showed that the sperm samples supplemented with 30 μg/mL PC had significantly higher motility, membrane integrity, acrosome integrity, and mitochondrial membrane potential compared with other groups after 48 h to 72 h storage, suggesting that PC had beneficial effects on sperm quality during chilling. Similar to this study, Wen et al. [19] found that the best concentration of PC for the preservation of goat semen at 4 °C was 30 μg/mL, while Li et al. [18] and Wang et al. [21] showed that PC improved sperm quality in the concentration of 50 μg/mL during liquid preservation of boar at 17 °C and cryopreservation of bull, respectively, and Wu et al. [20] showed that 10 μg/mL PC could enhance the motility and membrane integrity of post-thaw sperm in Hu sheep. The differences may be due to different species or different experimental conditions. In addition, their results also showed an increase in antioxidant enzyme activities, including SOD (Superoxide dismutase), CAT (Catalase), and GPx (Glutathione peroxidase), whereas a reduction in the levels of ROS and MDA. In this study, T-AOC and the expression levels of antioxidant-related genes (SOD1, CAT, and GPx1) in 30μg/mL PC-supplemented samples were significantly higher compared with those in control samples after 72 h storage at 4 °C, supporting that PC had a potential function on improving dog sperm antioxidant capacity during chilling.

Yang et al. [33] showed that procyanidin reduced the accumulation of ROS by regulating signaling pathways such as MAPK, NF-κB, Nrf2, and PI3K/Akt, thereby reducing oxidative stress (OS) damage to cells. Zhang et al. [34,35] studied the effect of procyanidin on oxidative damage of porcine granulosa cells and revealed that procyanidin could target FOXO1 and down-regulate the expression of Fas protein to reduce apoptosis and ROS levels. Li et al. [36], Rajput [37], and Li et al. [38] suggested that procyanidin may alleviate cellular oxidative damage by activating the Nrf2 signaling pathway. The above results are basically consistent with that described by Yang et al. [33]. Therefore, it can be speculated that procyanidin exerts an antioxidant effect on sperm through similar signaling pathways, but the specific molecular mechanism needs further study.

## 5. Conclusions

In conclusion, the present study found that procyanidin improved the quality of canine sperm stored at 4 °C. The results demonstrated that the addition of PC at 30 μg/mL may protect canine chilled sperm motility, membrane integrity, acrosome integrity, and mitochondrial membrane potential, as well as T-AOC and the expression levels of antioxidant-related genes. We believe that the antioxidant capability of PC plays a crucial role in improving sperm quality.

## Figures and Tables

**Table 1:** Primer sequence used for the analysis of gene expression.

Gene	Primer Sequence (5′–3′)	Product Size (bp)	NCBI Accession No.
ACTB	F: TGGCCGAGGACTTTGATTGT	171	NM_001195845.3
	R: CTCTTTCCCTCCCCTGTGTG		
SOD1	F: GTTGGAGACCTGGGCAATGT	211	NM_001003035.1
	R: TCCCAATGACACCACAAGCC		
CAT	F: CCTATCCTGACACTCACCGC	219	NM_001002984.1
	R: GCACATCTGGCGAACATTGG		
GPx1	F: CGGGCATCAGGAAAACGCTA	209	NM_001115119.1
	R: TGATGAACTTGGGGTCGGTC		

Abbreviations: F, forward; R, reverse; ACTB, actin beta; SOD1, superoxide dismutase 1; CAT, catalase; GPx1, glutathione peroxidase 1.

**Table 2:** Effects of different concentrations of procyanidin on dog sperm motility parameters after being stored at a cool temperature for 72 h.

Motility Parameters	Time (h)	Groups			
		Control	10 μg/mL PC	30 μg/mL PC	50 μg/mL PC
TM (%)	0	96.60 ± 0.24 ^Aa^	97.80 ± 0.58 ^Aa^	97.00 ± 0.63 ^Aa^	97.20 ± 0.37 ^Aa^
	24	93.20 ± 0.37 ^Bb^	95.80 ± 0.58 ^Aa^	96.20 ± 0.58 ^Aab^	96.00 ± 0.45 ^Aab^
	48	92.20 ± 0.58 ^Bb^	94.00 ± 0.55 ^Aab^	95.00 ± 0.32 ^Ab^	94.60 ± 0.51 ^Ab^
	72	89.00 ± 1.00 ^Bc^	91.00 ± 2.83 ^ABb^	94.80 ± 0.86 ^Ab^	91.80 ± 1.07 ^ABc^
VCL (um/s)	0	105.15 ± 2.78 ^Aa^	94.14 ± 4.20 ^Ba^	103.23 ± 2.05 ^ABa^	102.68 ± 3.97 ^ABa^
	24	86.29 ± 1.07 ^Ab^	77.24 ± 1.09 ^Cb^	82.83 ± 1.96 ^ABb^	81.94 ± 1.19 ^Bb^
	48	74.78 ± 0.72 ^Ac^	77.38 ± 2.77 ^Ab^	77.96 ± 0.51 ^Ab^	75.83 ± 0.36 ^Abc^
	72	74.34 ± 2.85 ^Ac^	77.84 ± 2.86 ^Ab^	77.08 ± 2.89 ^Ab^	73.39 ± 3.48 ^Ac^
VSL (um/s)	0	68.62 ± 1.03 ^Aa^	60.61 ± 3.06 ^Ba^	73.07 ± 2.07 ^Aa^	68.23 ± 3.00 ^Aa^
	24	64.78 ± 0.88 ^Ab^	56.62 ± 1.24 ^Bab^	57.76 ± 1.88 ^Bb^	58.61 ± 1.42 ^Bb^
	48	52.70 ± 0.22 ^Ac^	53.34 ± 0.71 ^Ab^	54.44 ± 0.69 ^Ab^	53.51 ± 0.71 ^Abc^
	72	51.53 ± 1.06 ^ABc^	55.11 ± 2.11 ^Aab^	46.91 ± 1.52 ^Bc^	49.27 ± 3.13 ^ABc^
VAP (um/s)	0	75.11 ± 1.17 ^ABa^	67.96 ± 3.52 ^Ba^	75.80 ± 1.21 ^Aa^	75.42 ± 3.25 ^ABa^
	24	65.55 ± 1.10 ^Ab^	61.04 ± 1.03 ^BCb^	59.90 ± 1.73 ^Cb^	64.13 ± 1.27 ^ABb^
	48	56.73 ± 0.45 ^BCc^	60.13 ± 0.88 ^Ab^	56.16 ± 0.49 ^Cbc^	58.22 ± 0.52 ^Bbc^
	72	57.80 ± 1.62 ^ABc^	60.24 ± 2.32 ^Ab^	52.74 ± 1.62 ^Bc^	53.99 ± 3.01 ^ABc^
LIN (%)	0	63.32 ± 0.71 ^ABa^	60.31 ± 1.89 ^Bb^	66.16 ± 0.46 ^Aa^	60.84 ± 0.54 ^Ba^
	24	64.83 ± 0.63 ^ABa^	65.13 ± 0.75 ^Aa^	62.77 ± 0.39 ^BCb^	61.91 ± 1.04 ^Ca^
	48	59.93 ± 1.14 ^Ab^	59.68 ± 1.13 ^Ab^	60.61 ± 0.33 ^Ac^	59.65 ± 1.18 ^Aa^
	72	48.74 ± 0.73 ^Bc^	57.01 ± 1.59 ^Ab^	56.42 ± 0.92 ^Ad^	54.53 ± 0.88 ^Ab^
WOB (%)	0	70.69 ± 0.47 ^Bb^	69.24 ± 1.09 ^Bb^	73.48 ± 0.38 ^Aa^	69.46 ± 0.77 ^Ba^
	24	74.41 ± 0.34 ^Aa^	72.79 ± 0.65 ^Ba^	71.52 ± 0.13 ^BCb^	70.71 ± 0.55 ^Ca^
	48	70.94 ± 0.33 ^Ab^	71.31 ± 0.53 ^Aab^	70.26 ± 0.23 ^ABc^	69.32 ± 0.69 ^Ba^
	72	61.67 ± 1.24 ^Bc^	65.84 ± 1.62 ^Ac^	65.94 ± 0.48 ^Ad^	62.44 ± 0.62 ^Bb^
STR (%)	0	88.39 ± 0.76 ^Aa^	85.89 ± 1.25 ^ABa^	88.28 ± 0.83 ^ABa^	85.48 ± 0.90 ^Ba^
	24	86.72 ± 0.52 ^Aa^	84.88 ± 1.17 ^ABa^	86.39 ± 0.49 ^ABb^	83.87 ± 1.01 ^Bab^
	48	82.34 ± 0.68 ^Cb^	84.31 ± 0.38 ^ABa^	84.48 ± 0.09 ^Ac^	82.73 ± 0.75 ^BCb^
	72	80.80 ± 0.36 ^Bb^	84.15 ± 0.49 ^Aa^	83.47 ± 0.58 ^Ac^	81.79 ± 0.65 ^Bb^
ALH (μm)	0	4.96 ± 0.23 ^Aa^	5.18 ± 0.18 ^Aa^	4.92 ± 0.06 ^Aa^	5.07 ± 0.12 ^Aa^
	24	4.38 ± 0.09 ^Aab^	4.58 ± 0.13 ^Aab^	4.73 ± 0.14 ^Aab^	4.54 ± 0.16 ^Ab^
	48	3.84 ± 0.08 ^Bbc^	4.26 ± 0.13 ^ABbc^	4.44 ± 0.09 ^Ab^	3.94 ± 0.06 ^Bc^
	72	3.27 ± 0.11 ^Cc^	3.70 ± 0.08 ^ABc^	3.94 ± 0.06 ^Ac^	3.41 ± 0.11 ^BCd^

Note: Values are expressed as mean ± SEM (*n* = 3). Different uppercase superscripts (A, B, and C) in the same row indicate significant differences (*p* < 0.05). Different lowercase superscripts (a, b, c, and d) in the same column indicate significant differences (*p* < 0.05). Abbreviations: TM, total motility; VCL, curvilinear velocity; VSL, straight-line velocity; VAP, average path velocity; LIN, linearity; WOB, wobble coefficient; STR, Straightness; ALH, amplitude of lateral head displacement.

**Table 3:** Effects of different concentrations of procyanidin on dog sperm plasma membrane integrity and acrosome membrane integrity after being stored at a cool temperature for 72 h.

Analysis	Time (h)	Groups			
		Control	10 μg/mL PC	30 μg/mL PC	50 μg/mL PC
Membrane integrity (%)	0 h	72.13 ± 0.04 ^Ba^	73.71 ± 0.49 ^ABa^	74.89 ± 0.70 ^Aa^	72.87 ± 0.72 ^Ba^
	24 h	65.45 ± 0.35 ^Cb^	67.76 ± 0.48 ^Bb^	70.94 ± 0.41 ^Ab^	69.87 ± 0.57 ^Ab^
	48 h	59.34 ± 0.32 ^Dc^	62.73 ± 0.23 ^Cc^	65.49 ± 0.29 ^Ac^	64.39 ± 0.29 ^Bc^
	72 h	54.12 ± 0.40 ^Cd^	56.83 ± 0.19 ^Bd^	59.83 ± 0.27 ^Ad^	57.63 ± 0.31 ^Bd^
Acrosome integrity (%)	0 h	74.97 ± 0.59 ^Aa^	73.69 ± 0.15 ^Aa^	74.10 ± 0.46 ^Aa^	73.48 ± 0.76 ^Aa^
	24 h	64.97 ± 0.56 ^Bb^	67.84 ± 0.25 ^Ab^	67.37 ± 0.31 ^Ab^	65.01 ± 0.24 ^Bb^
	48 h	60.12 ± 0.36 ^Cc^	64.34 ± 0.32 ^Ac^	64.61 ± 0.41 ^Ac^	62.77 ± 0.20 ^Bc^
	72 h	49.26 ± 0.38 ^Dd^	52.64 ± 0.23 ^Bd^	54.69 ± 0.23 ^Ad^	50.56 ± 0.56 ^Cd^

Note: Values are expressed as mean ± SEM (*n* = 3). Different uppercase superscripts (A, B, C, and D) in the same row indicate significant differences (*p* < 0.05). Different lowercase superscripts (a, b, c, and d) in the same column indicate significant differences (*p* < 0.05).

## Data Availability

The data presented in this study are available on request from the corresponding author.
